# Spatial distribution and correlation characteristics of heavy metals in the seawater, suspended particulate matter and sediments in Zhanjiang Bay, China

**DOI:** 10.1371/journal.pone.0201414

**Published:** 2018-08-02

**Authors:** Jibiao Zhang, Fengxia Zhou, Chunliang Chen, Xingli Sun, Yuzhen Shi, Hui Zhao, Fajin Chen

**Affiliations:** 1 College of Chemistry and Environmental Science, Guangdong Ocean University, Zhanjiang, China; 2 College of Ocean and Meterology, Guangdong Ocean University, Zhanjiang, China; 3 Guangdong Province Key Laboratory for Coastal Ocean Variation and Disaster Prediction Technologies, Guangdong Ocean University, Zhanjiang, China; CAS, CHINA

## Abstract

Concentrations of eight heavy metals (i.e., Fe, Mn, Cr, Ni, Cu, Zn, Cd and Pb) in the seawater, suspended particulate matter (SPM) and sediments of the Zhanjiang Bay were investigated in 2014. The concentrations of metals were generally low in the seawater and sediments of the Zhanjiang Bay in winter and summer, indicating good environmental quality in the bay. The distribution patterns of Fe and Mn in three phases indicated the influence of terrestrial inputs. The partition coefficients log(*K*_d_) between the dissolved and particulate phases showed a general decrease in the order of Pb≈Cd>Fe≈Mn>Ni≈Cr>Zn>Cu. The concentrations of some metals in the dissolved and particulate phases showed seasonal variations. Phytoplankton production and complexation reactions may contribute to this phenomenon. The relationships among metals in different phases were different, and there were few close relationships among metals in the dissolved phase, many close relationships in the particulate phase, and more close relationships in the sedimentary phase. This finding may be related to the different mobility levels of metals in different phases.

## Introduction

Of all organic and inorganic contaminants, heavy metals are of particular concern due to their environmental persistence, biogeochemical recycling and potential ecological risks. Many aquatic organisms assimilate dissolved metals directly, causing unwanted bioaccumulation. Particulate or sedimentary metals are easily assimilated and accumulated by filter-feeding organisms, especially filter-feeding bivalves [1]. Metals such as Cu and Zn are essential biological micronutrient elements that are required for the growth of many aquatic organisms, but these micronutrients can become toxic at high concentrations [2]. Other metals, such as Cr, Pb, and Cd, are not required for the growth of aquatic organisms and even trace amounts can be highly toxic to marine organisms [3,4].

In estuarine and coastal environments, heavy metals can be generally partitioned into dissolved, particulate and sedimentary phases. Heavy metals in different phases can also interact with each other. For example, dissolved metals can be transformed into the particulate phase through adsorption and flocculation [5]. Metals in the particulate phase can be desorbed from particulate matter into the water body or deposited into the sediment, while they can also be released back into the water column from the sediments by resuspension [6]. Heavy metals in different phases present different biogeochemical behaviors due to their different responses to environmental changes [7]. The processes of desorption, phytoplankton assimilation and redox conditions have profound influences on the physical and chemical behaviors of dissolved heavy metals [8,9]. Suspended particulate matter (SPM) has a high capacity to interact with a range of inorganic and organic contaminants through surface complexation, ligand exchange, hydrophobic association, and so on [6]. Thus, the content of SPM play an important role in marine environmental quality through affecting the concentration and distribution of heavy metals. Therefore, many marine environmental quality standards for heavy metals have been established in many countries to protect the marine environment [6,10,11]. Therefore, the study of the concentrations and distributions of heavy metals in seawater, SPM and sediment, the relationships among heavy metals in these different phases and related environmental parameters, are critically important.

Zhanjiang Bay (ZJB) is located in the Leizhou Peninsula of southern China and connects with the South China Sea (SCS). In recent decades, rapid economic growth and urban development have occurred in the area surrounding ZJB [12,13]. In 2014, the GDP of Zhanjiang was 2.3×10^3^ million yuan, which was 10% higher than that in 2013 [14]. The wastewater load discharged by Zhanjiang City in 2014 was 346 million tons [15]. According to the report of the Bulletin of Marine Environment Status of Guangdong Province in 2014, part of the seawater near Zhanjiang was polluted with high concentrations of phosphate and inorganic nitrogen [16]. ZJB is a complex region with respect to geography and hydrodynamics. The waters in ZJB are profoundly influenced by two water regimes: river discharge and oceanic water from the SCS. There are many sources of metals in ZJB, mainly including river runoff, wastewater discharge and atmospheric deposition. The dynamic variations and biogeochemical processes related to heavy metals in ZJB may cause important influences on environmental quality in the bay. Therefore, it is necessary to understand the spatial-temporal patterns of metals in ZJB. However, there have been few studies that have discussed the characteristics of the spatial-temporal variations of heavy metals in this area. In this study, eight major/trace metals (Fe, Mn, Cr, Ni, Cu, Zn, Cd and Pb) with important environmental significance [11,17,18] were investigated in ZJB in winter and summer of 2014. Through analyzing the distribution patterns of metals in three phases as well as the variations of water temperature, salinity, dissolved oxygen level, SPM concentrations and chlorophyll *a* (Chl *a*) in ZJB, we examined the possible relationships among different heavy metals and related environmental parameters to illuminate the possible behaviors and transition processes of heavy metals in different phases.

## Materials and methods

### Study area

**Fig 1:**
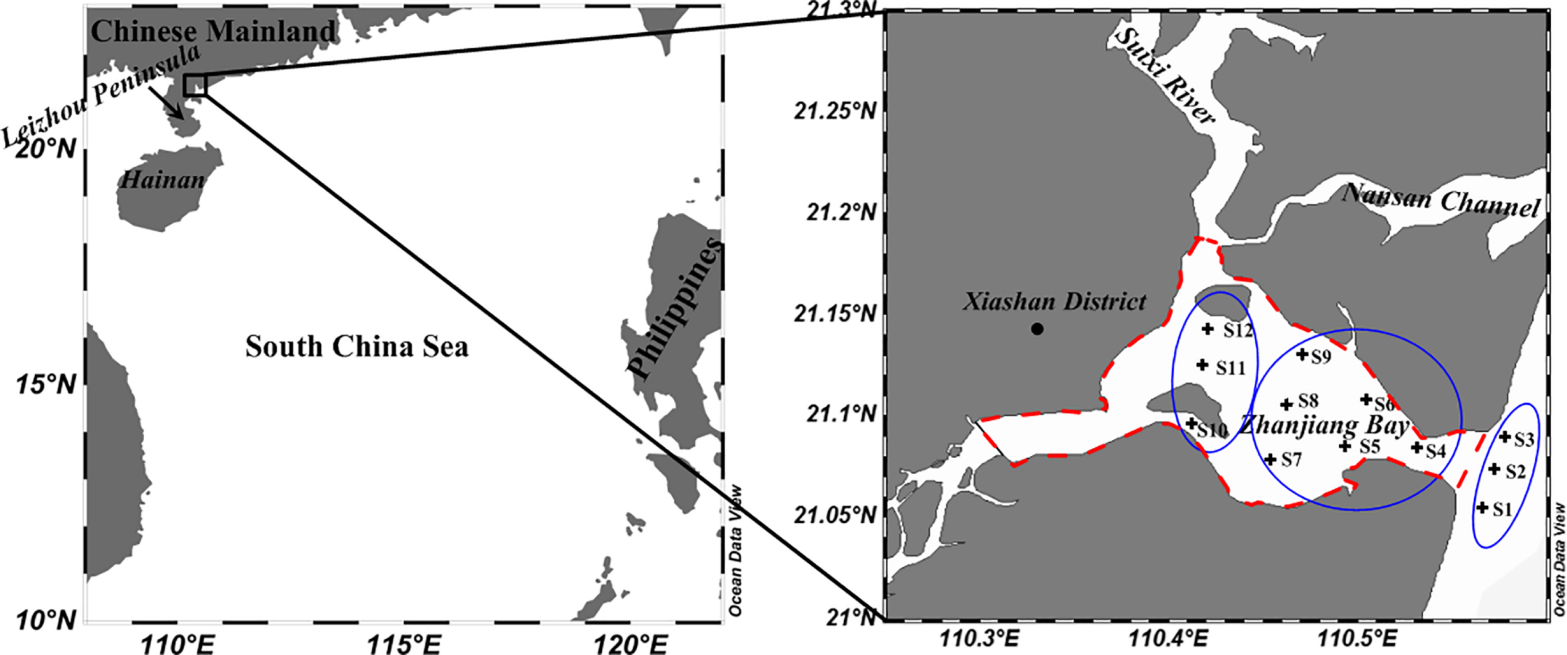
Study area, Zhanjiang Bay and sampling stations in ZJB.

ZJB is located in the northeast of the Leizhou Peninsula, which belongs to the southwest area of Guangdong Province (Fig 1), China. ZJB is a semi-closed and drowned valley lagoon with a narrow tidal entrance that is less than 2 km wide. The Suixi River is the main river that discharges into ZJB. The water area of this bay is approximately 190 km^2^, which is framed by the red dash lines in Fig 1. ZJB is a natural deep-water harbor. The normal waterway depth of ZJB ranges from 8 m to 28 m, and the deep-water channel between 26 m and 44 m is over 10 km long. ZJB lies in a subtropical monsoon climate zone with warm temperatures and high rainfall from April to September each year. The cool and dry season is from November to February.

### Sampling and pretreatment

Samples at twelve stations in ZJB (Fig 1) were collected in January (winter) and June (summer) 2014 for SPM and seawater. In consideration of more stable and minimal changes with seasons for sediments, surface sediments were sampled only once in January 2014. Two parallel water samples were collected manually at a depth of 0.5 m at each station. The samples were collected by using an in-house developed telescopic plastic barrel fitted with an adaptor into which a sampling bottle could be inserted. Each seawater sample was immediately filtered through acid-treated cellulose acetate filters with known weights (0.45 μm in pore size, 47 mm in diameter, Thermo Fisher, USA) by a polycarbonate-based filtration holder (Thermo Fisher Nalgene 300–4100, USA). The filtrate was collected in a precleaned 1 L polyethylene bottle and acidified to a pH of approximately 2 using high-purity HNO_3_. The sample bottles were sonicated in 30% HNO_3_, 30% HCl, and ultrapure water (UW) (Millipore Elix3+A10, 18.2 MΩ resistivity) for 3 h in a water bath that was kept at 60°C. At the end of this process, the bottles were thoroughly rinsed with UW, then dried in a vacuum drying oven. Each bottle was then double bagged in polyethylene bags and stored until use. The polycarbonate-based filtration holder was precleaned with 2% nitric acid and then rinsed with UW before use.

Surface sediments (upper 3 cm) were collected with a stainless-steel grab sampler and were placed in acid-cleaned polyethylene bags. The collected samples were stored in a cooler box with ice bags and then frozen at -20°C within 12 h until further treatments. The frozen samples were freeze-dried by a vacuum freeze dryer (Heto-Holten, LyoPro3000) and then ground to pass through a 200-mesh nylon sieve and kept in clean containers before further analysis.

### Metal analysis

The filters containing SPM were oven-dried at 105°C and weighed again to determine the amounts of SPM. Then, they were completely digested with a mixture of concentrated nitric, hydrofluoric acids and H_2_O_2_ (5:2:2) in a Teflon digestion tank by a microwave digester (CEM MARS, USA) using the temperature programming procedure recommended by the manufacturer. The extracts for metal analyses were diluted to a final weight of 50 g with deionized water in a PE plastic bottle. For surface sediment samples, three aliquots of ~200 mg (dry weight) were digested following the same procedure. To avoid possible contamination and decrease the blank value as much as possible, extreme care was taken during sample preparation, filtration and digestion; all concentrated acids that were used were of guaranteed reagent grade, and deionized water with a quality of at least 18 MΩ resistivity (Millipore Elix3+A10, USA) was used for washing and solution preparation; all other experimental accessories that were used were carefully cleaned (detergent, tap water, 10% nitric acid and deionized water).

The dissolved metal concentrations in filtered and acidified seawater were measured by ICP-MS (Agilent 7500Cx, USA) after being diluted 10 times with 2% nitric acid (volume ratio). To guarantee measurement quality, an internal standard sample (Part# 5183–4680, including Sc, Ge, Y, In, Tb and Bi, Agilent Technologies) and certified reference seawater with trace metals (GBW(E)-080040, from the Second Institute of Oceanography, SOA of China) were used to ensure acceptable results of the seawater samples. Metal concentrations of SPM and surface sediment in an aliquot of digest solution were also determined by ICP-MS, and the Chinese national reference material of GBW-07314 was used to control the analytical quality of the SPM and sediments. The concentrations in the analytical blanks, the results of the certified reference material analyses, and all of the recoveries shown in Table 1 confirmed that the data were reliable.

**Table 1:** Testing results of various quality-control measurements (n = 3).

	Unit	Fe	Mn	Cr	Ni	Cu	Zn	Cd	Pb
Filtrate blank	μg l^-1^	1.23±0.06	0.39±0.03	0.19±0.02	0.15±0.01	0.36±0.06	0.61±0.10	0.01±0.00	0.03±0.01
Filter blank	mg g^-1^	0.04±0.02	1.06±0.17	0.10±0.01	0.02±0.00	0.01±0.01	2.90±0.18	0.01±0.00	0.01±0.01
GBW(E) 080040	Reference value (μg l^-1^)	na	na	5.00±0.4	na	5.00±0.4	70.0±3	1.00±0.06	10.0±0.6
	Measured value (μg l^-1^)	na	na	5.04±0.28	na	5.06±0.62	72.8±2.2	0.97±0.14	9.82±0.68
	Recovery (%)	na	na	100.8±5.6	na	101.2±12.4	104.0±3.1	97±14	98.2±6.8
GBW-07314	Reference value (μg g^-1^)	(37.5±1.8)×10^3^	(0.96±0.04)×10^3^	86.0±4.0	34.3±4.0	31.0±4.0	87.0±2.0	0.20±0.04	25.0±4.0
	Measured value (μg g^-1^)	(38.2±2.2)×10^3^	(0.93±0.26)×10^3^	85.4±3.6	32.2±1.8	32.8±2.1	88.8±4.3	0.20±0.06	23.2±2.2
	Recovery (%)	101.9±5.9	96.9±27	99.3±4.2	93.9±2.3	105.8±6.8	102.1±4.9	100±30	92.8±8.8

na: not available.

### The analysis of other environmental factors

Temperature (T), salinity (S) and pH measurements were conducted *in situ* during the course of sampling. A salinometer (Thermo Fisher Eutech Salt 6+, USA) was used to measure the salinity of the seawater, and a pH meter (Thermo Fisher Orion Star A221, USA) was used for the pH measurement. The content of dissolved oxygen (DO) was determined by the typical iodometric titration within 24 h after the pretreated samples had been transported to the laboratory. For the determination of Chl *a*, one liter of seawater at each station was immediately filtered through a cellulose acetate membrane onboard, and the filters were stored at -20°C until analysis in the laboratory. The Chl *a* retained on the filters was extracted with 90% acetone and determined using a spectrophotometer (Shimadzu UV-2450, Japan).

### Methods of data statistics and analyses

The analytical and statistical method of data used in this work included some common softwares, such as Microsoft Office Excel, Golden Software Surfer and SPSS Statistics. Therein, through Microsoft Office Excel, the tables of different data were listed and the distributions of concentrations vs stations were also graphed in this work. The study area and sampling stations were illustrated by Golden Software Surfer in Fig 1. Through SPSS Statistics, the correlation analysis and principle component analysis were used to analyze the interrelationship of different types of data.

## Results

For the convenience of a clear description, we divided the study area into three regions. Three stations (S1, S2 and S3) at the mouth of the bay were classified as the bay mouth (Fig 1). Three stations (S10, S11 and S12) in the inner parts of the bay were classified as the inner bay (Fig 1). The other stations (S4-S9) represented the middle of the bay (Fig 1).

### Environmental background during the winter and summer

**Fig 2:**
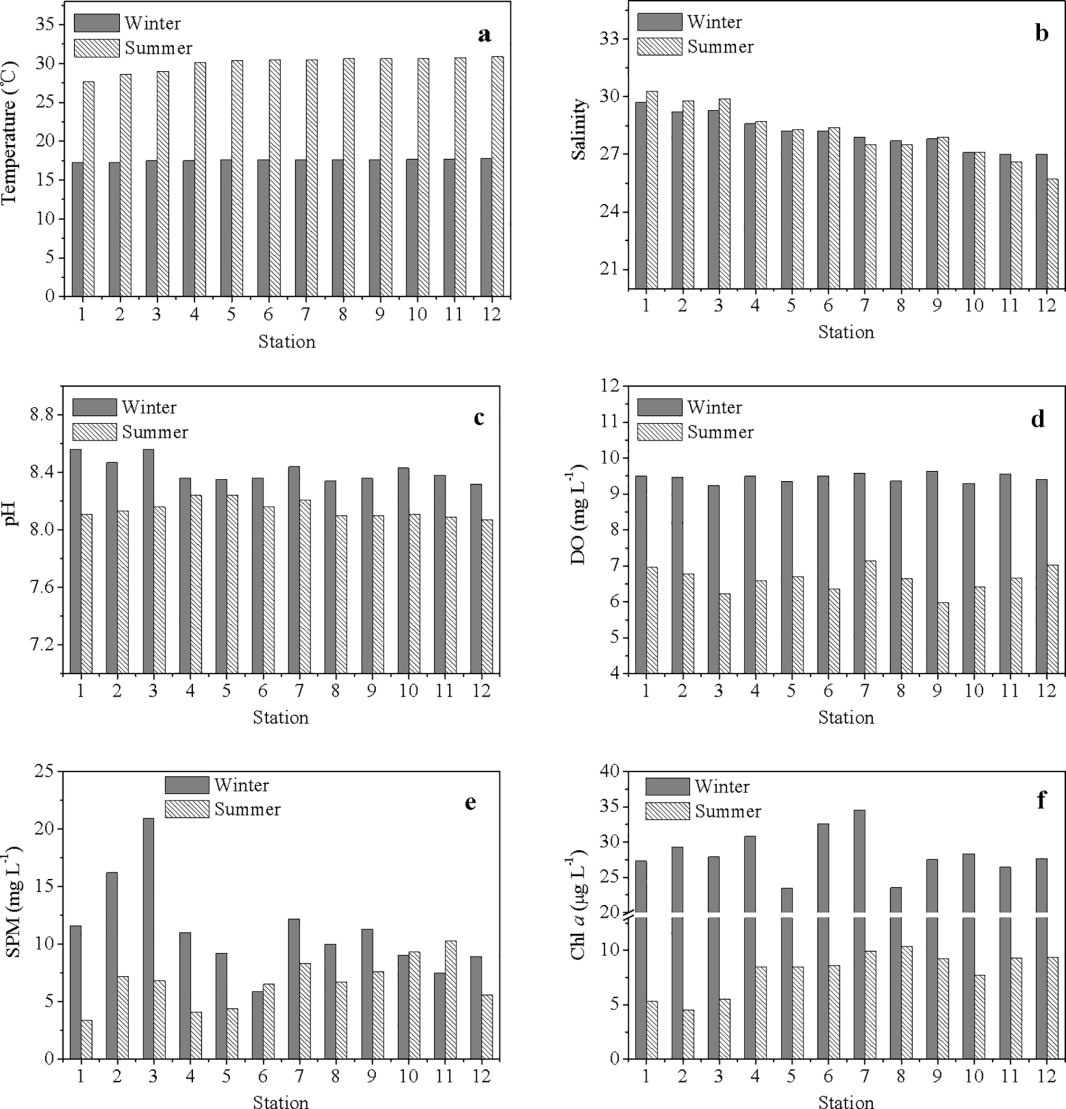
Spatial variations of environmental parameters in winter and summer. (a) Temperature; (b) Salinity; (c) pH; (d) DO; (e) SPM; (f) Chl *a*.

The temperature of the surface water in ZJB showed obvious seasonal differences. The mean water temperature in ZJB was 17.6°C in winter (range: 17.3~17.8°C) and 30.0°C in summer (range: 27.7~30.9°C) (Fig 2(A)). There were no obvious spatial variations in temperature in either season (Fig 2(A)). The salinity ranges in the water of ZJB ranged from 27.0 to 29.7 in winter and 25.7 to 30.3 in summer (Fig 2(B)). The salinity exhibited increasing tendencies from the inner bay to the bay mouth in both seasons (Fig 1; Fig 2(B)). The pH of the surface water was generally higher in winter than in summer, and the mean difference between the two seasons was 0.27. Unlike salinity, the spatial variations in pH were not obvious in either the winter or summer (Fig 2(B) and 2(C)). The DO concentrations of the surface water varied from 7.77 mg L^-1^ to 11.77 mg L^-1^ in winter and 5.45 mg L^-1^ to 8.53 mg L^-1^ in summer, which was indicative of well-oxygenated waters in this bay (Fig 2(D)). The range of DO concentrations in ZJB was comparable with that in other estuaries or bays with fewer pollutants [8].

The SPM concentrations in ZJB ranged from 5.9~20.9 mg L^-1^ in winter (average: 11.1 mg L^-1^) and 3.4~10.3 mg L^-1^ in summer (average: 6.7 mg L^-1^) (Fig 2(E)). In winter, the SPM concentrations in the bay mouth were higher than those in the middle bay and the inner bay (Fig 2(E)). However, this phenomenon was not shown in summer (Fig 2(E)). The average concentrations of Chl *a* in winter and summer were 28.3 and 8.08 μg L^-1^, respectively (Fig 2(F)), indicating more phytoplankton in winter than in summer.

### Heavy metals in different phases

#### Heavy metals in seawater

The concentrations of heavy metals in the seawater of ZJB are listed in Table 2. Among the eight studied metals, the mean concentrations in the two seasons decreased in the order of Fe, Zn, Mn, Cu, Cr, Ni, Pb, and Cd (Table 2). According to the National Standard of China for Seawater Quality (SWQ) of GB 3097–1997 [19], the seawater was classified into four levels (i.e., Grades I-IV) corresponding to different function zones, and these levels have already been used to evaluate the seawater quality in China. The mean concentrations of dissolved Zn, Cu, Cr, Ni, Pb and Cd in ZJB were all within the ranges of SWQ Grade I (Table 2), indicating that the water of ZJB was not polluted by these metals.

**Table 2:** The summary of heavy metal concentrations (μg L^-1^) in the seawater of ZJB and other coastal areas. The National Standard of China for Seawater Quality Guidelines are also shown for comparison.

Names of areas	Period		Fe	Mn	Cr	Ni	Cu	Zn	Cd	Pb	References
Zhanjiang Bay	Jan. 2014	Range	60.28–96.96	3.05–5.89	1.84–3.33	0.94–2.76	3.54–7.03	5.71–13.69	0.12–0.30	0.05–0.75	our results
		Mean	76.58	4.26	2.64	1.70	4.80	8.54	0.15	0.21	
	Jun. 2014	Range	65.53–108.4	3.56–7.54	1.49–4.25	0.46–1.55	3.35–5.24	9.97–25.70	0.04–0.14	0.09–0.51	our results
		Mean	84.06	4.76	2.77	0.90	4.00	16.74	0.08	0.25	
	2014	Range	60.28–108.4	3.05–7.54	1.49–4.25	0.46–2.76	3.35–7.03	5.71–25.70	0.04–0.30	0.05–0.75	our results
		Mean	80.32	4.51	2.70	1.30	4.40	12.64	0.12	0.23	
Bohai Bay	2004	Mean	na^a^	na	na	na	16.3	38.7	0.79	27.17	[20]
Jiaozhou Bay		Mean	na	na	na	na	3.48	48.93	0.13	22.72	[21]
Yellow River Estuary and adjacent sea	May 2009	Range	na	na	na	na	0.01–4.46	12.00–81.41	0.10–3.22	0.22–1.33	[22]
		Mean	na	na	na	na	2.65	37.67	0.68	0.51	
Major river estuaries of East-Hainan	2006–2007	Range	2.23–535.7	na	na	0.16–0.71	0.15–1.21	na	0.002–0.01	0.002–0.18	[23]
Pearl River Estuary	2009	Range	na	0.12–91.2	na	na	0.44–2.73	na	na	na	[24]
Yangtze River Estuary	Nov. 1998	Range	6.06–35.86	na	na	1.03–1.65	1.22–1.68	1.00–1.79	0.002–0.04	0.48–0.70	[25]
Yangtze River Estuary and Hangzhou Bay	2006	Mean	na	na	na	na	1.99	6.1	0.39	0.9	[26]
Scheldt Estuary	1995	Range	na	na	na	na	0.5–1.6	3.2–12.5	0–0.15	0.03–0.3	[8]
MCH Coastal Lagoon	2004–2006	Range	nd^b^-227	na	nd-35.2	nd-79.8	nd-1000	nd-1002	nd-7.1	nd-850	[4]
Grade I^c^			na	na	≤50	≤5	≤5	≤20	≤1	≤1	[19]
Grade II^c^			na	na	≤100	≤10	≤10	≤50	≤5	≤5	[19]
Grade III^c^			na	na	≤200	≤20	≤50	≤100	≤10	≤10	[19]
Grade IV^c^			na	na	>200	>20	>50	>100	>10	>10	[19]

^a^ na: not available.

^b^ nd: not detected.

^c^ Grades I-IV: the National Standard of China for Seawater Quality GB 3097–1997.

For comparison purposes, the eight heavy metal concentrations in seawater reported in other coastal areas are also listed in Table 2. The mean concentrations of the metals in ZJB were generally within the ranges reported in other coastal areas, as shown in Table 2.

Compared with the concentrations reported for the seawater from the Bohai Bay [20], the average concentrations of Cu, Zn, Cd and Pb recorded in ZJB were generally lower. The average concentrations of Cu and Cd in the seawater of ZJB were only comparable with those in the Jiaozhou Bay [21]. The average concentrations of Fe, Cu, Zn and Cd in ZJB were higher than only those in the Yangtze River Estuary [24,25]. Some of the metal concentrations in ZJB showed obvious seasonal variations. The mean Zn concentration was obviously higher in summer, and the mean concentrations of Ni and Cd were obviously higher in winter (Table 2). The mean concentrations of Fe, Mn, Cr, Cu and Pb exhibited no obvious seasonal variations, and their seasonal differences were generally within 20% (Table 2).

**Fig 3:**
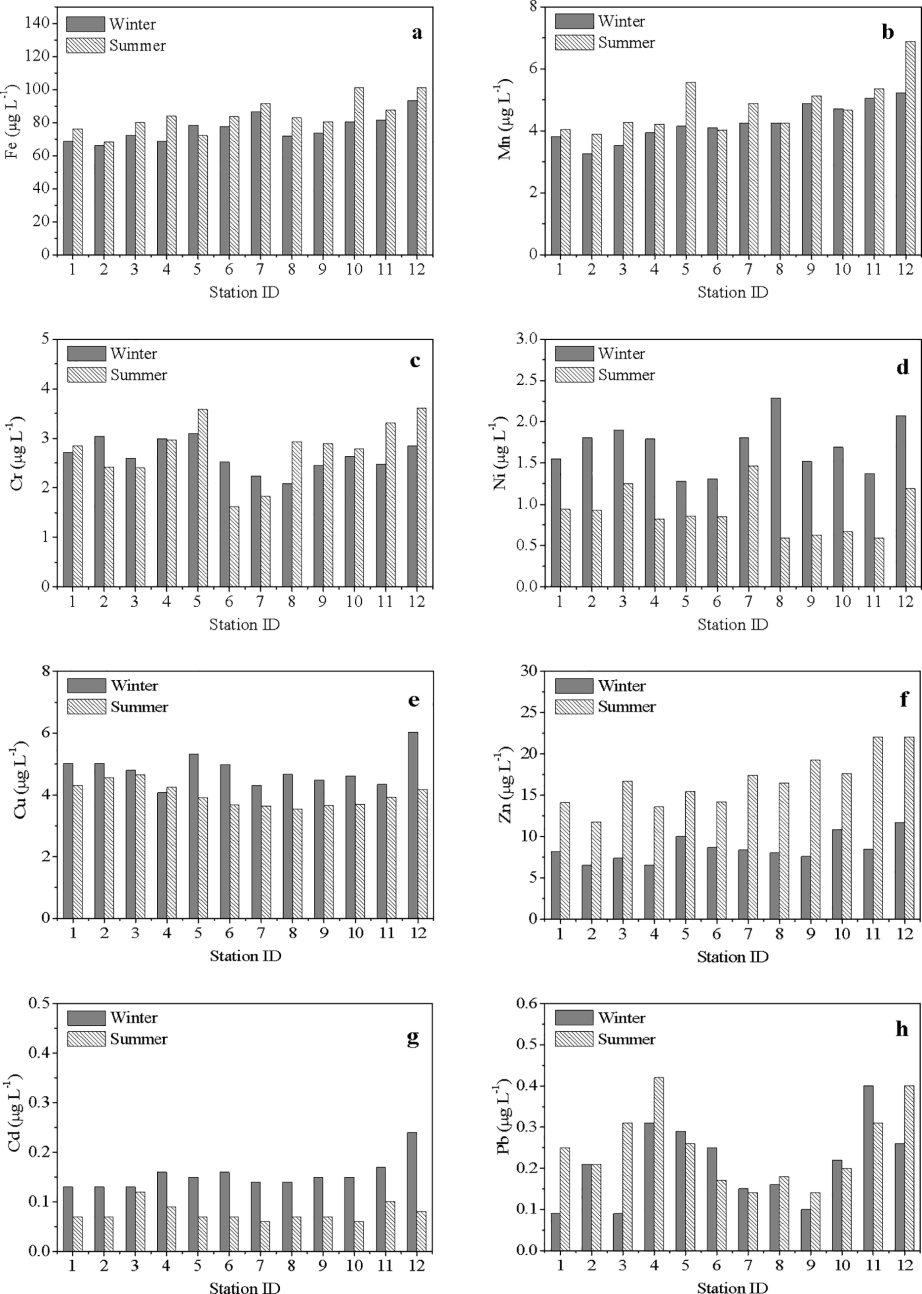
Spatial variations of heavy metals in seawater in winter and summer. (a) Fe; (b) Mn; (c) Cr; (d) Ni; (e) Cu; (f) Zn; (g) Cd; (h) Pb.

Fig 3 shows the spatial variations of dissolved metals in winter and summer in ZJB. The concentrations of Fe and Mn in both seasons generally decreased from the inner bay to the bay mouth (Fig 3(A) and 3(B); the concentrations of Zn in summer and Cd in winter presented distribution patterns that were similar to those of Fe and Mn (Fig 3(F) and 3(G); the concentrations of Zn in winter, Cd in summer, and other metals did not show obvious spatial distribution patterns (Fig 3). The results may indicate that the main sources for Fe and Mn in ZJB were terrigenous, and the sources of the other dissolved metals may be more complicated.

#### Heavy metals in suspended particulate matter

The concentrations of the eight heavy metals (Fe, Mn, Cr, Ni, Zn, Cu, Cd and Pb) in the suspended particulate matter in ZJB are presented in Table 3. The mean concentrations of Fe, Mn, Cr, Ni, Zn, Cu, Cd and Pb in the two seasons decreased in sequence. The mean concentrations of all other particulate metals except Fe, Cr and Ni were within the range reported in the other coastal areas listed in Table 3. The mean concentration of Fe recorded in this study was lower than the concentrations in the Bahía Blanca Estuary [27] and the major river estuaries in the East Hainan Island [23] (Table 3). The mean concentrations of Cr and Ni in this study were higher than those in the Yellow River Estuary [28] (Table 3).

**Table 3:** The summary of heavy metal concentrations (μg g^-1^ dry weight for all elements except when otherwise noted) in suspended particulate matter of ZJB and other coastal areas.

Names of areas	Period		Fe^a^	Mn^a^	Cr	Ni	Cu	Zn	Cd	Pb	References
Zhanjiang Bay	Jan. 2014	Range	10.54–41.26	0.34–2.21	73.31–281.4	58.96–188.7	15.60–47.17	120.5–482.6	11.96–39.54	40.70–86.27	our results
		Mean	22.07	0.89	160.76	112.47	27.14	296.9	23.55	56.28	
	Jun. 2014	Range	4.06–19.38	0.32–0.90	92.30–256.2	42.95–150.6	41.06–153.3	179.9–490.0	21.06–56.70	26.30–73.16	
		Mean	8.93	0.52	154.5	85.78	80.38	254.55	35.01	43.11	
	2014	Range	4.06–41.26	0.32–2.21	73.31–281.4	42.95–188.7	15.60–153.33	120.5–490.0	11.96–56.70	26.30–86.27	
		Mean	15.50	0.70	157.62	99.13	53.76	275.71	29.28	49.70	
Major river estuaries of East-Hainan	2006–2007	Range	31.81–106.0	na^b^	na	18.78–76.31	15.88–56.52	na	0.19–0.75	22.79–66.30	[23]
Yellow River Estuary	2013	Range	na	na	58.6–83.9	27.5–40.6	26.1–37.8	70.2–96.6	0.23–0.30	28.4–36.7	[27]
		Mean	na	na	65.4	32.7	32.4	81.1	0.27	31.9	
Scheldt Estuary	1995	Range	na	na	na	na	10–120	100–750	0.5–11	40–220	[8]
	May 1982	Range	na	na	na	na	31–314	58–608	4.4–35	88–355	[3]
		Mean	na	na	na	na	176	343	19	223	
Bahía Blanca Estuary	2011–2013	Range	22–96	0.63–1.50	na	na	26–241	na	nd-9.7	na	[28]

^a^ mg g^-1^ dry weight.

^b^ na: not available.

**Fig 4:**
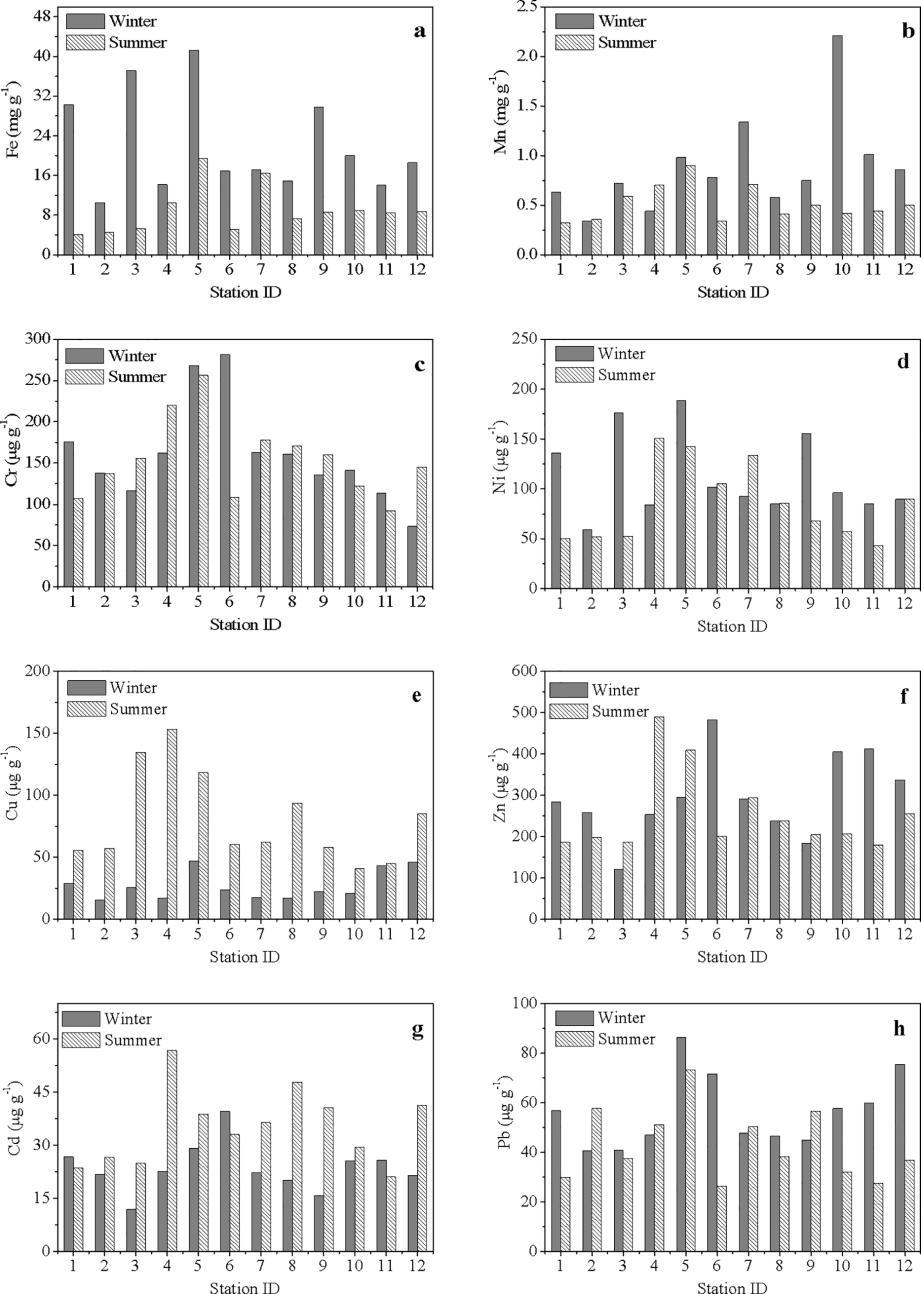
Spatial variations of heavy metals in suspended particulate matter in winter and summer. (a) Fe; (b) Mn; (c) Cr; (d) Ni; (e) Cu; (f) Zn; (g) Cd; (h) Pb.

Most of the metals in SPM presented strong seasonal variations. The mean concentrations of Fe, Mn, Ni, Zn and Pb in SPM were obviously higher in winter than in summer, while Cu and Cd showed the reverse pattern (Table 3). Unlike the distribution patterns of some metals that showed decreasing tendencies from the inner bay to the bay mouth in dissolved phase (Fig 3(A), 3(B), 3(F) and 3(G)), most of the metals in the particulate phase showed no obvious spatial patterns (Fig 4). This phenomenon suggested that the sources and/or behaviors of metals in the particulate phase may be different from those in the dissolved phase to some extent.

#### Heavy metals in surface sediments

Heavy metal concentrations in the surface sediments of ZJB were measured during the winter survey. The mean concentrations of the heavy metals in surface sediments decreased from Fe to Mn, Zn, Cr, Pb, Ni, Cu and then to Cd (Table 4). The National Standard of China for Marine Sediment Quality (MSQ) of GB 18668–2002 [35] is widely used to judge the potential risks of metals in marine sediments [11]. This standard classifies marine sediments into three classes based on the function and protection targets of the marine area. The mean concentrations of Zn, Cr, Pb, Cu and Cd in ZJB sediments were all within the range for MSQ Grade I, indicating that these metals were within good levels (Table 4), and the surface sediments in ZJB did not suffer from metal contamination.

**Table 4:** The summary of heavy metal concentrations (μg g^-1^ dry weight for all elements except when otherwise noted) in the surface sediments of ZJB and other coastal zones. The average upper continental crust values and related sediment quality guidelines are also shown for comparison purposes.

Name of areas	Period		Fe^a^	Mn^a^	Cr	Ni	Cu	Zn	Cd	Pb	References
Zhanjiang Bay	Jan. 2014	Range	34.20–42.33	0.32–0.55	38.72–88.34	17.28–26.12	8.50–30.06	40.37–121.4	0.12–0.20	27.61–58.18	our results
		Mean	38.28	0.42	63.83	22.43	18.74	73.60	0.15	43.89	
Eastern continental shelf, Hainan	2012–2014	Range	na^b^	na	32.4–73.9	11.0–35.5	13.7–45.0	37.8–114.3	0.07–0.35	11.1–31.1	[29]
		Mean	na	na	57.3	25.6	29.4	81.4	0.19	19.2	
Daya Bay	Dec. 2008	Range	20.7–33.6	0.5–1.0	42.1–127	10.7–31.0	2.35–48.7	53.2–114	na	18.6–47.3	[30]
		Mean	29.5	0.8	75.6	24.7	12.7	94.4	na	32.7	
Western Xiamen Bay	2004–2005	Mean	na	na	75	37.4	44	139	0.33	50	[31]
Coastal Bohai Bay	May 2008	Range	na	na	60.1–224.5	23.4–52.7	20.1–62.9	55.3–457.3	0.12–0.66	20.9–66.4	[17]
		Mean	na	na	101.4	40.7	38.5	131.1	0.22	34.7	
Pearl River Estuary	1999	Range	na	na	58.1–117.8	18.1–72.7	11.2–75.7	28.4–237.2	na	49.3–78.9	[32]
		Mean	na	na	89.0	41.7	46.2	150.1	na	59.3	
	Dec. 2008	Range	32.4–41.3	0.6–1.4	74.1–123	21.9–46.5	18.9–87.2	100–289	na	40.9–92.4	[30]
		Mean	37.5	0.9	106	36.7	45.7	177	na	57.9	
Yangtze River Estuary	Feb. 2011	Range	na	0.40–1.42	50.35–123.1	19.92–42.91	9.67–49.21	46.52–126.7	0.07–0.71	14.8–45.09	[33]
		Mean	na	0.77	79.1	31.9	24.7	82.9	0.19	23.8	
Upper continental crust (UCC)		Mean	35	0.6	35	20	25	71	0.098	20	[34]
Grade I^c^			na	na	≤80	na	≤35	≤150	≤0.5	≤60	[35]
Grade II^c^			na	na	≤150	na	≤100	≤350	≤1.5	≤130	[35]
Grade III^c^			na	na	≤270	na	≤200	≤600	≤5	≤250	[35]

^a^ mg g^-1^ dry weight.

^b^ na: not available.

^c^ Grades I-III: the National Standard of China for Marine Sediment Quality of GB 18668–2002.

For comparison purposes, the mean concentrations of heavy metals in the upper continental crust (UCC) and those of surface sediments reported in some coastal areas are also shown in Table 4. In the surface sediments of ZJB, the mean concentrations of Cr, Cd and Pb were clearly higher than those in the UCC (Table 4). Compared with those reported in coastal Bohai Bay and western Xiamen Bay, which are surrounded by heavily urbanized zones in China, the mean concentrations of Cr, Ni, Cu, Zn and Cd were lower in ZJB. The mean concentration of Pb that was recorded in this study was higher than the concentrations in the Daya Bay, the Yangtze River Estuary, coastal Bohai Bay and the eastern continental shelf of Hainan Island; however, the concentrations in this study were lower than those in western Xiamen Bay and the Pearl River Estuary.

**Fig 5:**
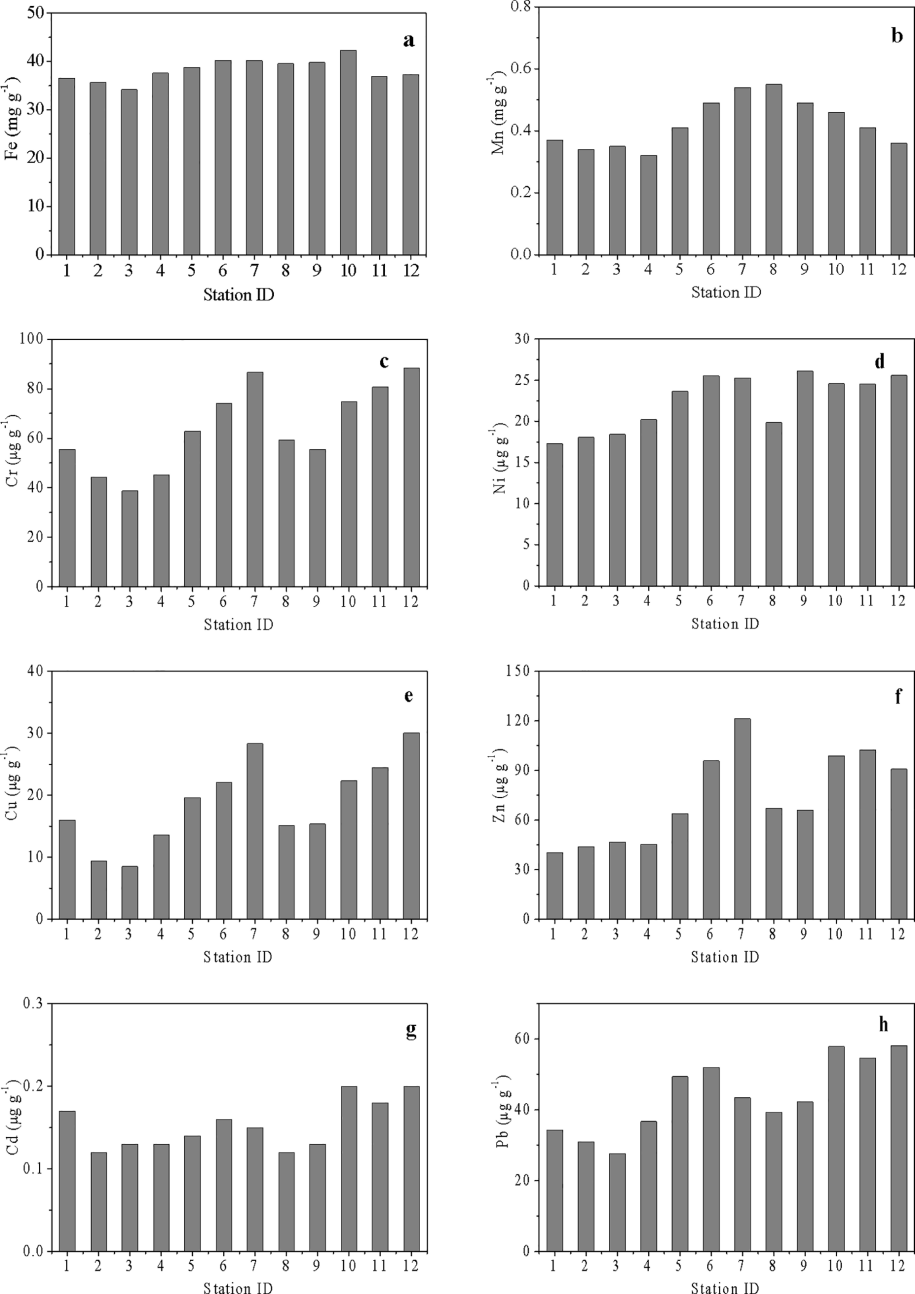
Spatial variations of heavy metals in surface sediments in winter and summer. (a) Fe; (b) Mn; (c) Cr; (d) Ni; (e) Cu; (f) Zn; (g) Cd; (h) Pb.

Fig 5 shows the spatial distributions of heavy metals in the surface sediments of ZJB. For most of the heavy metals in ZJB, their concentrations were generally low in the bay mouth compared with those in the middle bay and the inner bay, implying the potential influence of terrestrial inputs (Fig 5).

#### Partitions of heavy metals between dissolved and particulate phases

The existing speciation of metal elements in seawater systems is impacted by many environmental parameters, including temperature, salinity, pH, and SS [36,37]. The value of log(*K*_d_) is suitable for evaluating the partitioning balance of heavy metals between dissolved and suspended phases. The partition coefficient (*K*_d_) is defined as the ratio of the particulate metal concentration (μg g^-1^) to the dissolved metal concentration (mg L^-1^) [38–41]. A higher log(*K*_d_) value indicates a stronger affinity between the metal and suspended particles, and a lower log(*K*_d_) value means more metal exists in the dissolved phase.

Based on the data of both winter and summer, the mean values of log(*K*_d_) for the metals in ZJB followed the variation in the order of Pb≈Cd>Fe≈Mn>Ni≈Cr>Zn>Cu (Table 5). This result suggested that among the eight metals in ZJB, Pb and Cd were most strongly bound to SPM, while Cu and Zn were least partitioned into the particulate phase. The different partition behaviors are determined by the specific physical and chemical characteristics of the metals [39,41].

**Table 5:** The summary of partitioning coefficients (log(*K*_*d*_)) in ZJB and other areas in the world.

Name of areas	Period		Fe	Mn	Cr	Ni	Cu	Zn	Cd	Pb	References
Zhanjiang Bay	Jan. 2014	Range	5.15–5.67	4.99–5.68	4.41–5.05	4.47–4.97	3.49–3.95	4.15–4.61	4.95–5.37	5.32–5.95	our results
		Mean	5.32	5.21	4.73	4.69	3.67	4.40	5.14	5.60	
	Jun. 2014	Range	4.71–5.25	4.83–5.18	4.46–5.01	4.62–5.23	4.03–4.51	3.81–4.55	5.29–5.79	5.02–5.59	our results
		Mean	4.93	4.99	4.73	4.94	4.22	4.12	5.60	5.31	
	2014	Range	4.71–5.67	4.83–5.68	4.41–5.05	4.47–5.23	3.49–4.51	3.81–4.61	4.95–5.79	5.02–5.95	our results
		Mean	5.13	5.10	4.73	4.82	3.95	4.26	5.37	5.45	
Jiaozhou Bay, China		Range	na^a^	na	na	na	3.8–4.9	1.9–4.7	2.2–5.0	2.8–5.4	[42]
Estuaries of Hainan Island	2006–2007	Mean	6.0	na	na	na	4.8	na	4.8	na	[23]
North Australian Coastal and Estuaries	1996–2000	Range	5.7–8.8	na	na	3.8–5.3	3.7–5.4	4.4–6.7	3.3–6.3	5.5–7.2	[38]
Atlantic Ocean	1990	Range	na	na	na	na	4.5–4.9	na	5.1–6.2	4.7–6.4	[43]
Six Texas estuaries		Range	4.7–7.2	na	na	na	3.0–5.1	3.8–6.0	na	3.8–6.8	[37]
Scheldt Estuary, Belgium		Range	na	na	na	na	4.3–5.4	4.5–5.0	5.5–6.4	5.4–6.0	[8]

^a^ na: not available.

The high particle reactivities of Pb and Cd promote the association of these two metals with particulate matter, which lead to a higher value of log(*K*_d_). On the other hand, the low particle reactivity and stronger potential to form stable organic complexes allows Zn and Cu to more easily remain in the dissolved phase [41]. The partition coefficients (log(*K*_d_)) in ZJB were roughly of the same order of magnitude as those in other estuaries or bays throughout the world (Table 5). Compared with Jiaozhou Bay in China [42], the accumulation abilities of Zn, Cd and Pb in SPM seem to be much stronger in ZJB.

**Fig 6:**
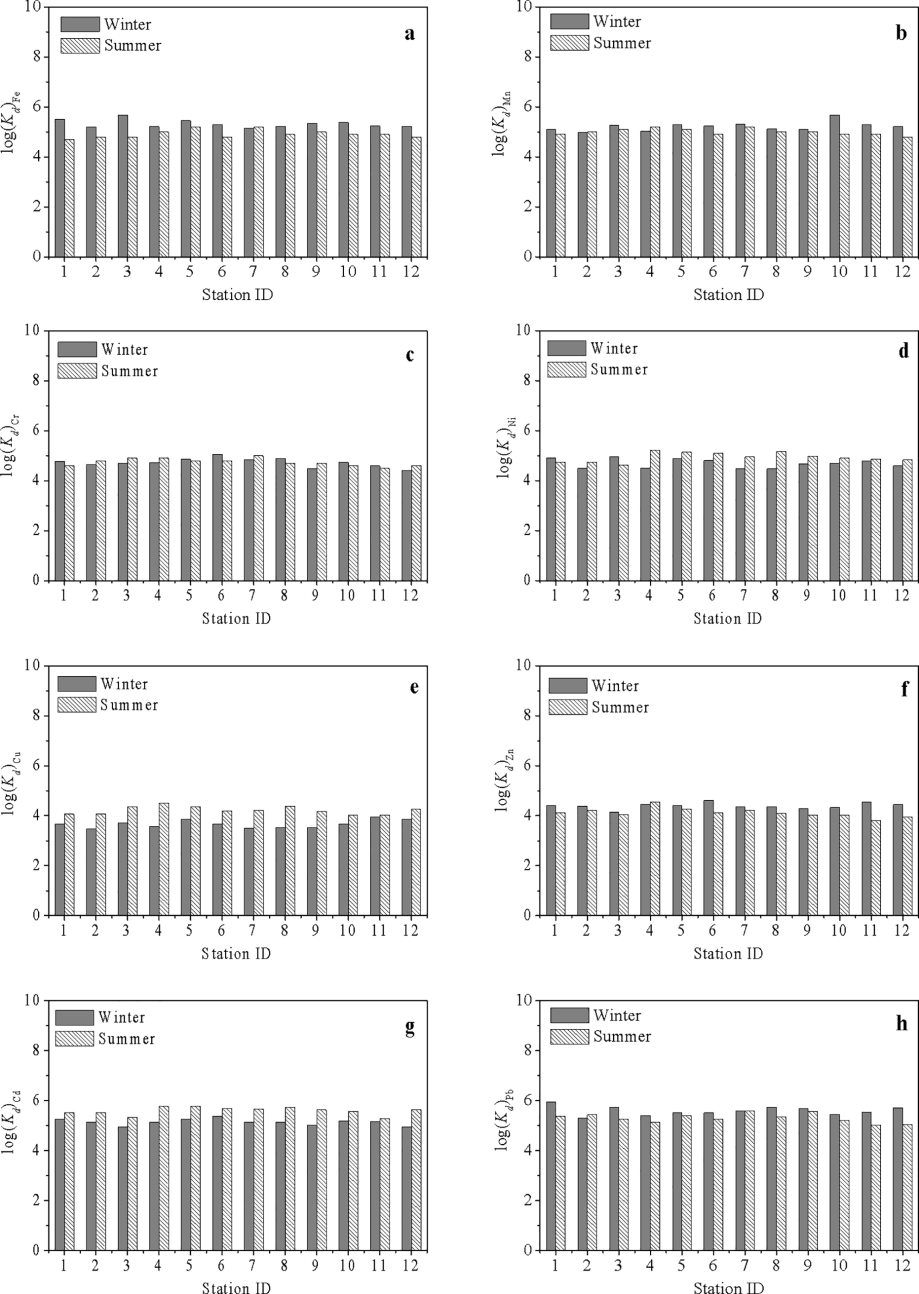
Spatial variations of log(*K*_d_) between dissolved and particulate phases in winter and summer. (a) Fe; (b) Mn; (c) Cr; (d) Ni; (e) Cu; (f) Zn; (g) Cd; (h) Pb.

Fig 6 shows the spatial variations of log(*K*_d_) for different metals in winter and summer. There were no obvious spatial variations in the values of log(*K*_d_) for all metals in both seasons (Fig 6). However, some seasonal variations could be seen in the log(*K*_d_) values of some metals. The log(*K*_d_) values were generally higher in winter than in summer for Fe, Zn and Pb, and the reverse was true for Cu and Cd.

## Discussion

### Correlation analysis

In a complex marine system, variations of any environmental factors is not independent it will be interdepend with other environmental parameters, which can be analyzed by correlation method. Correlation analysis is based on Pearson or Spearman product moment coefficients and the corresponding correlation results can be presented in covariance correlation matrices. The covariance is a measure of this relationship and depends on the variability of each of the two variables. Correlation analysis can estimate the strength of the relationship between any pair of variables [44]. For normal environmental factors in the ZJB water, all of pH, DO, SPM and Chl *a* showed significant negative correlations with temperature; Chl *a* showed significant positive correlations with pH, DO and SPM (Table 6). As belonging to a tropical marine system, the water temperature of ZJB in winter is rather warm (with an average value of 17.6°C) and suitable for algal growth [45,46]. In such a warm water, marine phytoplankton can utilize more CO_2_ and produce more O_2_ [47,48], which may contribute to generally high pH and DO in winter in ZJB (Fig 2(C) and 2(D)). Meanwhile, the concentrations of Chl *a* in the water of ZJB were generally higher in winter (Fig 2(F)).

**Table 6:** Pearson correlation matrix for metals in different phases and related environmental parameters.

		T	S	pH	DO	SPM	Chl *a*	Fe	Mn	Cr	Ni	Cu	Zn	Cd	Pb
	S	-0.075													
	pH	-0.887*	0.221												
	DO	-0.978*	-0.024	0.876*											
	SPM	-0.572*	0.110	0.677*	0.553*										
	Chl *a*	-0.951*	-0.071	0.875*	0.961*	0.590*									
(a) Dissolved (n = 24)	Fe	**0.436**	-0.702*	**-0.499**	-0.370	-0.304	-0.316	1							
	Mn	0.368	-0.757*	**-0.467**	-0.288	**-0.422**	-0.292	0.641*	1						
	Cr	0.148	-0.196	-0.165	-0.120	-0.108	-0.118	0.063	**0.411**	1					
	Ni	-0.829*	0.034	0.761*	0.827*	0.570*	0.777*	-0.280	-0.241	-0.283	1				
	Cu	-0.716*	0.202	0.586*	0.663*	0.308	0.575*	-0.257	-0.241	0.055	0.639*	1			
	Zn	0.878*	-0.386	-0.857*	-0.853*	**-0.506**	-0.812*	0.659*	0.644*	0.301	-0.715*	-0.561*	1		
	Cd	-0.863*	0.118	0.657*	0.829*	0.349	0.816*	-0.163	-0.105	-0.040	0.735*	0.788*	-0.641*	1	
	Pb	0.186	-0.250	-0.315	-0.176	**-0.504**	-0.216	0.289	0.391	**0.476**	-0.232	0.027	0.270	0.092	1
(b) Particulate (n = 24)	Fe	-0.635*	0.028	0.717*	0.651*	0.527*	0.601*	1							
	Mn	**-0.451**	-0.281	**0.467**	0**.449**	0.068	**0.478**	**0.417**	1						
	Cr	-0.051	0.129	0.132	0.066	-0.277	0.090	0.319	0.167	1					
	Ni	-0.293	0.034	**0.490**	0.339	0.220	0.304	0.827*	0.302	**0.491**	1				
	Cu	0.684*	0.096	-0.547*	-0.713*	-0.612*	-0.711*	-0.360	-0.185	0.260	0.053	1			
	Zn	-0.199	0.209	0.240	0.241	**-0.405**	0.269	0.086	0.515*	0.559*	0.255	0.207	1		
	Cd	0.582*	-0.179	**-0.471**	-0.543*	-0.665*	**-0.467**	0.341	-0.106	0.523*	0.097	0.678*	**0.505**	1	
	Pb	**-0.424**	-0.037	0.365	**0.415**	-0.116	0.380	0.529*	**0.457**	0.583*	**0.454**	-0.037	0.616*	0.129	1
(c) Sedimentary (n = 12)	Fe	0.275	**-0.585**	-0.490	0.160	**-0.651**	0.160	1							
	Mn	0.363	-0.447	-0.321	0.238	-0.388	0.077	0.753*	1						
	Cr	-0.021	-0.765*	-0.443	0.237	**-0.681**	0.207	0.526	0.451	1					
	Ni	0.397	-0.801*	**-0.647**	0.343	**-0.636**	0.212	**0.653**	0.491	0.770*	1				
	Cu	-0.013	-0.732*	-0.457	0.245	**-0.675**	0.229	0.493	0.359	0.983*	0.772*	1			
	Zn	0.231	-0.769*	-0.385	0.235	-0.547	0.365	**0.610**	**0.601**	0.915*	0.810*	0.868*	1		
	Cd	-0.187	-0.532	-0.076	-0.056	-0.514	0.074	0.273	-0.044	0.731*	0.464	0.740*	0.565	1	
	Pb	0.095	-0.859*	**-0.628**	0.058	-0.814*	-0.036	**0.604**	0.295	0.851*	0.827*	0.842*	0.760*	0.768*	1

Bold values are significant, and stars (*) are strongly significant.

#### Relationships between dissolved metals and environmental factors

In the ZJB water, many metals in the dissolved phase showed significant correlations with many environmental parameters (Table 6). Fe showed a significant positive correlation with temperature and significant negative correlations with salinity and pH. Mn showed significant negative correlations with salinity, pH and SPM. No significant correlations were found between Cr and other environmental parameters. Ni showed significant negative correlations with temperature and significant positive correlations with pH, DO, SPM and Chl *a*. Cu showed a significant negative correlation with temperature and significant positive correlations with pH, DO and Chl *a*. Significant positive correlations between dissolved Cu and DO have also been observed in other estuaries or bays [3,8]. Zn showed a significant positive correlation with temperature and significant negative correlations with pH, DO, SPM and Chl *a*. The correlations between Cd and other environmental parameters were similar to those of Cu. Pb showed a significant negative correlation with only SPM.

Different correlations between dissolved metals and related environmental parameters result from various behaviors, and/or different sources and/or sinks of metals. Salinity had significant negative correlations with Fe and Mn, indicating that terrestrial inputs strongly contributed to the distributions of Fe and Mn in the ZJB water. Ni, Cu and Cd in the water showed significant positive correlations with pH, DO and Chl *a*. These metals may have close relationships with phytoplankton production. High primary production usually leads to high concentrations of Chl *a* and DO and high pH. DOC concentrations may also increase during this course of phytoplankton growth [49]. Some metals have complexation properties with organic matter, which could keep these metals in the dissolved phase[8]. Therefore, significant positive correlations were presented between these three metals (i.e., Ni, Cu and Cd) and pH, DO and Chl *a*. Compared with Ni, Cu and Cd, Zn had reverse relationships with pH, DO and Chl *a*. Bruland and Lohan [50] reported that Zn was a nutrient-type metal. Therefore, the enrichment of Zn in phytoplankton may contribute to the significant negative correlations between Zn and pH, DO and Chl *a*. The relationships between temperature and Ni, Cu, Zn and Cd may be influenced by the variations in Chl *a* to some extent. In the ZJB water, the mean temperature of 17.6°C in winter was suitable for the rapid growth of phytoplankton, resulting in relatively high concentrations of Chl *a* and DOC (Fig 2(A) and 2(F)) [43,44]. Concentrations of dissolved Ni, Cu and Cd increased in winter due to their complexation action with organic matter, as discussed above. Meanwhile, the high concentrations of Chl *a* in winter were in favor of the enrichment of Zn by phytoplankton [2]. As a result, Ni, Cu and Cd had significant negative correlations with temperature, and Zn had significant positive correlations with temperature (Table 5). The significant negative correlations between Pb and SPM may be associated with the high concentrations of SPM, which can absorb more dissolved Pb due to the high availability of SPM adsorption surfaces [8].

Due to the various responses of dissolved metals to the environmental conditions, some of the correlations were different among eight metals with each other in the ZJB water (Table 6). The reverse responses of Zn and Ni, Cu and Cd to the phytoplankton production, as discussed above, may account for the significant negative correlations between Zn and Ni, Cu and Cd. Although Zn exhibited different behaviors with Fe and Mn, significant positive correlations were found between Zn and Fe, Zn and Mn, which may indicate that these metals may have similar behaviors in other aspects [41]. This result needs to be further explored in future studies.

#### Relationships among particulate metals and environmental parameters

In the ZJB environments, the relationships between particulate metals and environmental parameters were different from those between dissolved metals and environmental parameters to some extent (Table 6). Particulate Fe had a significant negative correlation with temperature and significant positive correlations with pH, DO, SPM and Chl *a*, which may imply that high primary production was in favor of the enrichment of Fe in SPM [51]. Although the behavior of particulate Mn was roughly similar to that of particulate Fe to some extent, it was evidently different that no significant correlation existed between particulate Mn and SPM, compared with the significant correlation between particulate Fe and SPM. The different absorption and desorption behaviors of Fe and Mn (Table 6), which could be inferred from the relationships between dissolved Fe, Mn and SPM, may be responsible for this phenomenon. Similarly, the behaviors of dissolved and particulate Cr did not show significant correlations with the related environmental parameters. Particulate Ni showed a significant positive correlation with only pH, which may be caused by the increased absorption ability by SPM in high pH environments. Particulate Cu showed a significant positive correlation with temperature and significant negative correlations with pH, DO, SPM and Chl *a*. The complexation of Cu with dissolved organic matter, which could be deduced from the relationships between dissolved Cu and related environmental parameters, contributed to the negative correlations between particulate Cu and some environmental parameters [12]. A similar phenomenon could also be seen for particulate Cd.

Generally, similar behaviors or sources of the metals could be concluded by the significant positive relationships between the metals in the particulate phase [5]. Significant positive relationships could be observed between particulate Pb and particulate Fe, Mn, Cr, Ni and Zn, which probably suggested their similar behaviors or sources in the particulate phase in ZJB [5]. Although significant positive relationships were also observed between particulate Cu and Cd, these two particulate metals were not significantly correlated with the other metals in the particulate phase, which indicated that the behaviors or the sources of particulate Cu and Cd were different from the other particulate metals. The complexation of particulate Cu and Cd with dissolved organic matter, as discussed above, may be the main reason that led to their different behaviors from other particulate metals [4].

#### Relationships among sedimentary metals and environmental parameters

Generally, salinity [52] can reflect the influence of terrestrial input to a certain degree in coastal regions and bays, and the SPM could ultimately settle on surface sediments. Therefore, the above two parameters (i.e., salinity and SPM) presented significant correlations with most of the sedimentary metals in ZJB (Table 6), where the sedimentary metals in the nearshore areas were generally terrigenous, and the SPM was the main source of surface sediments [11,28]. It was an interesting finding that the SPM concentrations generally showed significant negative correlations with most sedimentary metals (Table 6). This phenomenon may be related to the size compositions of the SPM and the hydrodynamic conditions, which need further study in the future. Compared with the metals in dissolved and particulate phases, sedimentary metals seemed to be less influenced by many water environmental parameters, such as T, pH, DO and Chl *a*, as inferred from the relatively few significant correlations between sedimentary metals and these water environmental parameters (Table 6).

#### Relationships among partition coefficients and related parameters

The log(*K*_d_) coefficient can be regarded as a way to evaluate the partitioning ability of heavy metals between dissolved and suspended/sediment phases; a higher log(*K*_d_) value indicates a stronger affinity between metals and suspended particles, and a lower log(*K*_d_) value means more metals exist in the dissolved phase [38,39]. Table 7 shows the correlations among the partition coefficients of heavy metals and the related environmental parameters. Significant positive correlations (Table 7) were found among the partition coefficients of Fe, Mn, Zn and Pb, indicating that the partition behaviors are similar between dissolved and particulate phases for these metals. The partition coefficients of Cu and Cd were negatively correlated with the partition coefficients of Fe, indicating that the partition behaviors of Cu and Cd were different from that of Fe. The partition behaviors of Ni were also similar to those of Cu and Cd, which was inferred from the significant positive correlations between their partition coefficients.

**Table 7:** Pearson correlation matrix for partition coefficients and related environmental parameters.

	log(*K*_*d*_)_Fe_	log(*K*_*d*_)_Mn_	log(*K*_*d*_)_Cr_	log(*K*_*d*_)_Ni_	log(*K*_*d*_)_Cu_	log(*K*_*d*_)_Zn_	log(*K*_*d*_)_Cd_	log(*K*_*d*_)_Pb_
log(*K*_d_)_Fe_	1							
log(*K*_d_)_Mn_	0.672*	1						
log(*K*_d_)_Cr_	0.169	0.265	1					
log(*K*_d_)_Ni_	-0.109	-0.185	0.177	1				
log(*K*_d_)_Cu_	-0.599*	-0.390	0.094	0.717*	1			
log(*K*_d_)_Zn_	0.560*	0.562*	0.324	-0.232	**-0.438**	1		
log(*K*_d_)_Cd_	-0.591*	**-0.475**	0.317	0.716*	0.829*	-0.343	1	
log(*K*_d_)_Pb_	0.719*	**0.437**	0.138	-0.242	-0.550*	**0.470**	-0.522*	1
T	-0.755*	-0.617*	0.019	0.617*	0.874*	-0.686*	0.874*	-0.643*
S	0.016	-0.104	0.300	-0.054	-0.040	0.156	-0.025	0.229
pH	0.874*	0.643*	0.134	-0.348	-0.751*	0.662*	-0.722*	0.671*
DO	0.770*	0.591*	-0.037	-0.583*	-0.878*	0.695*	-0.833*	0.628*
SPM	0.607*	0.229	-0.169	-0.345	-0.659*	0.039	-0.704*	**0.409**
Chl *a*	0.760*	0.609*	0.004	-0.537*	-0.870*	0.669*	-0.800*	0.585*

Bold values are significant, and stars (*) are strongly significant.

Based on the results in Table 7, a higher temperature seems to help the desorption of Fe, Mn, Zn and Pb from SPM, which agrees well with the general adsorption rule of physical chemistry [53]. However, for Ni, Cu and Cd, the correlation variations between their log(Kd) values and temperature presented reverse rules. The relatively lower concentrations of the three metals may be the possible cause. Except for Cr, the partition coefficients of all metals were significantly correlated with many environmental parameters (i.e., pH, DO, SPM, and Chl *a*) (Table 7), suggesting that the above environmental factors may probably regulate the partition behaviors of metals between seawater and SPM in ZJB. High phytoplankton primary production usually leads to high concentrations of Chl *a*, DO, and SPM and high pH [8]. According to the correlation analysis among the partition coefficients of heavy metals and the environmental parameters, we could deduce that high primary production could cause Fe and Pb to be more easily partitioned into the particulate phase and could cause Cu and Cd to be more easily partitioned into the dissolved phase. Similar conclusions were also obtained in other studies [3,8,54]. Similar with Fe and Pb to some extent, high concentrations of Chl *a*, DO and high pH were favorable for the partitioning of Mn and Zn to into the particulate phase. However, high concentrations of Chl *a* and DO seemed to be favorable for the partitioning of Ni into the dissolved phase.

### Principal component analysis

Principal component analysis (PCA) is a multivariate exploratory technique with two main applications: reducing the number of variables and detecting relationships among them [55,56]. This method was used to identify principal components from three groups of parameters in ZJB: dissolved metals and environmental parameters (Group 1), particulate metals and the related environmental parameters (Group 2), and sedimentary metals and the environmental parameters in the water column (Group 3) (Table 8).

**Table 8:** Loadings of experimental variables on significant principal components for the datasets from ZJB.

Group 1	Principal Component			Group 2	Principal Component			Group 3	Principal Component		
	1	2	3		1	2	3		1	2	3
T	**-0.967**	-0.201	-0.026	T	**-0.950**	0.069	0.098	T	0.184	**0.715**	-0.185
S	0.250	**-0.811**	**0.438**	S	0.027	-0.139	**0.717**	S	**-0.882**	-0.027	0.293
pH	**0.932**	-0.006	-0.033	pH	**0.935**	0.011	0.136	pH	**-0.658**	-0.258	0.364
DO	**0.938**	0.270	-0.031	DO	**0.954**	-0.037	-0.144	DO	0.271	0.101	**0.653**
SPM	**0.664**	-0.124	-0.341	SPM	**0.661**	**-0.538**	0.236	SPM	**-0.810**	0.089	0.135
Chl *a*	**0.913**	0.271	-0.111	Chl *a*	**0.938**	-0.023	-0.195	Chl *a*	0.152	0.089	0.860
D-Fe	**-0.543**	**0.577**	**-0.400**	P-Fe	**0.799**	0.212	0.365	S-Fe	**0.735**	0.396	0.001
D-Mn	**-0.530**	**0.731**	-0.125	P -Mn	**0.533**	0.389	**-0.418**	S-Mn	**0.560**	**0.618**	0.107
D-Cr	-0.241	**0.423**	**0.568**	P -Cr	0.121	**0.808**	0.303	S-Cr	**0.923**	-0.251	0.135
D-Ni	**0.851**	0.200	-0.166	P -Ni	**0.487**	**0.498**	**0.496**	S-Ni	**0.905**	0.180	0.047
D-Cu	**0.707**	0.255	0.351	P -Cu	**-0.688**	**0.484**	0.249	S-Cu	**0.904**	-0.287	0.141
D-Zn	**-0.927**	0.178	-0.106	P -Zn	0.196	**0.825**	-0.357	S-Zn	**0.897**	0.031	0.236
D-Cd	**0.785**	**0.487**	0.127	P -Cd	**-0.559**	**0.711**	-0.020	S-Cd	**0.634**	**-0.660**	-0.027
D-Pb	-0.320	**0.532**	**0.617**	P -Pb	**0.469**	**0.688**	-0.015	S-Pb	**0.926**	-0.229	-0.232
Total variance	53.3%	18.3%	9.9%	Total variance	44.5%	23.6%	10.7%	Total variance	52.9%	12.9%	11.4%
Cumulative variance	53.3%	71.6%	81.4%	Cumulative variance	44.5%	68.1%	78.7%	Cumulative variance	52.9%	65.8%	77.2%

Note: Bold values indicate strong loadings; D-dissolved; P-particulate; S-sedimentary.

Based on the principal component extraction from the PCA, the first three principal components were extracted from each group of parameters (Table 8). For the metals in the dissolved phase and the related environmental parameters, three principal components (PC1-PC3) were identified that accounted for 81.4% of the total data variance. The PC1 of this group, accounting for 53.3% of the data variance, had high positive loadings for temperature, pH, DO, SPM, Chl *a*, Ni, Cu and Cd and high negative loadings for Fe, Mn and Zn. The results indicated that PC1 had biological characteristics, and primary production (i.e., temperature, pH, DO, SPM and Chl *a*) in PC1 favored the remainder of Ni, Cu and Cd in the dissolved phase and the clearance of Fe, Mn and Zn from the water. The PC2 of this group, accounting for 18.3% of the data variance, had high positive loadings for Fe, Mn, Cr, Cd, and Pb and had a high negative loading for salinity. This component may represent the hydrological characteristics of these parameters, which indicate that Fe, Mn, Cr, Cd and Pb in the dissolved phase may be influenced by terrestrial inputs to some extent [39,57]. The PC3 of this group, accounting for 9.9% of the data variance, had high positive loadings for salinity, Cr, and Pb and a high negative loading for Fe. This component may represent the influence of the outer seawater on the behaviors of dissolved Fe and Cr in ZJB. Considering the distribution patterns and seasonal variations of the studied metals in the dissolved phase and the related environmental parameters, we obtained the conclusion that the seasonal variations of most of the studied dissolved metals in ZJB were mainly influenced by phytoplankton primary production, and terrestrial inputs could have some effects on the spatial variations of some dissolved metals in ZJB.

For the metals in the particulate phase and the related environmental parameters, three principal components (PC1-PC3) were also identified that accounted for 78.7% of the total data variance. The PC1 of this group, accounting for 44.5% of the data variance, had high positive loadings for temperature, pH, DO, SPM, Chl *a*, Fe, Mn, Ni and Pb and high negative loadings for Cu and Cd. Like the PC1 of Group 1, the PC1 of Group 2 also had biological characteristics. Phytoplankton production seemed to favor the enrichment of Fe, Mn, Ni and Pb in the particulate phase and to induce the dissociations of Cu and Cd from the particulate phase. The PC2 of Group 2, accounting for 23.6% of the data variance, had high positive loadings for Cr, Ni, Cu, Zn, Cd and Pb and a negative loading for SPM. This result may indicate that low concentrations of SPM were favorable for the enrichment of metals in the particulate phase. The possible reason is because the particle size is generally smaller in a low concentration of SPM and shows a stronger adsorbability for inorganic and organic pollutants in water body. The PC3 of Group 2, accounting for 10.7% of the data variance, had high positive loadings for salinity and Ni and a negative loading for Mn. Different absorption and/or desorption behaviors of Mn and Ni may be responsible for this phenomenon (Table 5).

For the metals in the sedimentary phase and the environmental parameters of the water body, three principal components (PC1-PC3) were identified that accounted for 77.2% of the total data variance. The PC1 of this group, accounting for 52.9% of the data variance, had high positive loadings for all studied sedimentary metals and high negative loadings for salinity, pH and SPM. Considering the distribution patterns of sedimentary metals and the related environmental parameters, we concluded that all studied metals in the sediments were mainly influenced by terrestrial inputs. For the PC2 and PC3 of this group, there were few high loadings of sedimentary metals, reflecting lower disturbance of the water environment on the distribution of metals in surface sediments.

### Relationships among metals in different phases

Generally, the relationships among different metals are interdependent in different phases, and it’s complicated between metals and related environmental parameters. As revealed in Table 6, many water parameters (such as temperature, pH, DO, and Chl *a*) had close relationships with metals in dissolved and particulate phases in ZJB. These environmental parameters in the water seemed to have little influence on the sedimentary metals, as revealed by the poor correlations among them (Table 6). However, the salinity and SPM of the water environments seemed to have close relationships with many heavy metals in the surface sediments (Table 6). The settlement of SPM and the resuspension of surface sediment may strongly contribute to the close relationships between SPM and sedimentary heavy metals. The significant correlations between salinity and many sedimentary heavy metals seemed to be a coincidence, as both the salinity of the water and sedimentary heavy metals were mainly controlled by terrestrial input [41,57]. The relationships among metals in different phases were also different, and there were few close relationships among metals in the dissolved phase, many close relationships among metals in the particulate phase, and closer relationships among metals in the sedimentary phase, which may be attributed to the fact that metals in the dissolved phase more easily migrated and could also strongly interact with the water environment; the mobilities of particulate metals were relatively weak, and their interactions with the water environment were not as close as those of dissolved metals; the mobility of sedimentary metals was weakest, and their interactions with the water environment were less weak than those of dissolved and particulate metals.

Metals in dissolved and particulate phases generally had reverse correlation relationships with water environmental parameters [57–59]. For example, dissolved Cu had a negative correlation with temperature and positive correlations with pH, DO and Chl *a*, while particulate Cu had a positive correlation with temperature and negative correlations with pH, DO and Chl *a*. The reason for this difference may be that Cu in dissolved and particulate phases exhibited reverse behaviors in response to the variations in environmental parameters. There were also some metals in dissolved and particulate phases that had similar relationships with environmental parameters like Cu. For example, both dissolved and particulate Ni had positive correlations with pH, and both dissolved and particulate Zn had negative correlations with SPM. Other processes such as terrestrial inputs or sediment release may contribute to that phenomenon.

## Conclusions

The environmental conditions in Zhanjiang Bay that were inferred from the survey of eight heavy metals (Fe, Mn, Cr, Ni, Cu, Zn, Cd and Pb) were found to be in good conditions due to the low concentrations of these metals in both the dissolved and sedimentary phases. There were obvious seasonal variations in the dissolved Zn, Ni and Cd (i.e., water phase) and the particulate Fe, Mn, Ni, Zn, Pb, Cu and Cd (i.e., particulate phase). The distribution patterns of some metals in the dissolved and sedimentary phases indicated the potential influence of terrestrial inputs. The partition coefficients log(*K*_*d*_) between dissolved and particulate phases showed a general decrease in the order of Pb≈Cd>Fe≈Mn>Ni≈Cr>Zn>Cu. The values of log(*K*_*d*_) in some of the eight metals presented obvious seasonal variations.

Correlation and principal component analyses indicated that both terrestrial inputs and biological processes regulated the distributions and seasonal variations in metals in the three different phases. Dissolved Fe and Mn were mainly influenced by terrestrial inputs, while dissolved Ni, Cu, Zn and Cd were mainly influenced by biological processes. For the metals in the particulate phase, biological processes seemed to be the main factor that controlled the behaviors of most of the metals in ZJB. For the metals in the sedimentary phase, all metals were mainly influenced by terrestrial inputs. Phytoplankton production in ZJB could cause Fe, Pb, Mn and Zn to more easily enter the particulate phase, while it could cause Cu, Cd and Ni to more easily enter the dissolved phase.

Metals in the different phases interact with the water environments with different intensities, resulting in many strong correlations in sedimentary metals, relatively weaker correlations in particulate metals, and the weakest correlations in dissolved metals.

## Supporting information

S1 Fig Spatial variations of environmental parameters in winter and summer. (a) Temperature; (b) Salinity; (c) pH; (d) DO; (e) SPM; (f) Chl *a*.
(XLS)

S2 Fig Spatial variations of heavy metals in seawater in winter and summer. (a) Fe; (b)Mn; (c) Cr; (d) Ni; (e) Cu; (f) Zn; (g) Cd; (h) Pb.
(XLS)

S3 Fig Spatial variations of heavy metals in suspended particulate matter in winter and summer. (a) Fe; (b)Mn; (c) Cr; (d) Ni; (e) Cu; (f) Zn; (g) Cd; (h) Pb.
(XLS)

S4 Fig Spatial variations of heavy metals in surface sediments in winter and summer. (a) Fe; (b)Mn; (c) Cr; (d) Ni; (e) Cu; (f) Zn; (g) Cd; (h) Pb.
(XLS)

S5 Fig Spatial variations of log(*K*_d_) between dissolved and particulate phases in winter and summer. (a) Fe; (b)Mn; (c) Cr; (d) Ni; (e) Cu; (f) Zn; (g) Cd; (h) Pb.
(XLS)
